# Deep reinforcement learning-based pairwise DNA sequence alignment method compatible with embedded edge devices

**DOI:** 10.1038/s41598-023-29277-6

**Published:** 2023-02-16

**Authors:** Aryan Lall, Siddharth Tallur

**Affiliations:** Department of Electrical Engineering (EE), IIT Bombay, Mumbai, 400076 India

**Keywords:** Genome informatics, Electrical and electronic engineering

## Abstract

Sequence alignment is an essential component of bioinformatics, for identifying regions of similarity that may indicate functional, structural, or evolutionary relationships between the sequences. Genome-based diagnostics relying on DNA sequencing have benefited hugely from the boom in computing power in recent decades, particularly due to cloud-computing and the rise of graphics processing units (GPUs) and other advanced computing platforms for running advanced algorithms. Translating the success of such breakthroughs in diagnostics to affordable solutions for low-cost healthcare requires development of algorithms that can operate on the edge instead of in the cloud, using low-cost and low-power electronic systems such as microcontrollers and field programmable gate arrays (FPGAs). In this work, we present EdgeAlign, a deep reinforcement learning based method for performing pairwise DNA sequence alignment on stand-alone edge devices. EdgeAlign uses deep reinforcement learning to train a deep Q-network (DQN) agent for performing sequence alignment on fixed length sub-sequences, using a sliding window that is scanned over the length of the entire sequence. The hardware resource-consumption for implementing this scheme is thus independent of the lengths of the sequences to be aligned, and is further optimized using a novel AutoML based method for neural network model size reduction. Unlike other algorithms for sequence alignment reported in literature, the model demonstrated in this work is highly compact and deployed on two edge devices (NVIDIA Jetson Nano Developer Kit and Digilent Arty A7-100T, containing Xilinx XC7A35T Artix-7 FPGA) for demonstration of alignment for sequences from the publicly available Influenza sequences at the National Center for Biotechnology Information (NCBI) Virus Data Hub.

## Introduction

Genomic medicine has the potential to make disease detection more efficient and cost-effective^1^. Genetics will be crucial not just in determining the cause of a disease, but also in determining how a person responds to various treatments. Nanopore sequencing is a unique, scalable technology that enables direct, real-time analysis of long DNA or RNA fragments. Oxford Nanopore Technologies MinION^2^ is one such portable nanopore sequencing device that can be easily operated in the field with features including monitoring of run progress and selective sequencing. Data collected through such devices are typically stored and analysed on the cloud, or desktop-grade servers in well-equipped laboratories. To truly harness the potential of genome-based diagnostics for affordable healthcare solutions, that do not rely on expensive cloud storage and connectivity (e.g. for resource-constrained communities in low and middle income countries), it is necessary to develop efficient methods for analysing the data and delivering insights with automated and easy-to-implement workflows integrated with portable edge-devices. One such data analysis requirement is DNA sequence alignment, an essential component of bioinformatics and computational genomics for comparing two or more sequences, in order to identify regions of similarity that may be a consequence of functional, structural or evolutionary relationships between the sequences.

Several heuristic methods and algorithms have been proposed for the sequence alignment problem, of which the Needleman–Wunsch (NW) algorithm^3^ is one of the earliest and most commonly used method for pairwise sequence alignment. However, the computational complexity of such a dynamic programming-based alignment approach is proportional to the product of the lengths of the two DNA sequences, and is therefore challenging to be implemented on hardware devices with limited memory and computation blocks. In a multiple sequence alignment (MSA) problem, several sequences are aligned simultaneously. Clustal W^4^ is a well-known tool for MSA. It uses the progressive alignment method and determines the best alignment by matching sequences that are most similar first, then moving on to sequences that are least similar. Several implementations have also been proposed for improving this sequence alignment method including a few multi-thread based implementations on graphics processing units (GPUs)^5–7^ for improving the throughput. Pairwise alignment is commonly performed using various tools such as banded alignment, BLAST, MUMmer^8–10^ etc. along with various cloud processing solutions^11^. BLAST^12^ is the most commonly used tool, which is also available to be downloaded and run on a local computer, or an edge-computing platform running an operating system such as NVIDIA Jetson Nano. BLAST is also often used as part of other algorithms that require approximate sequence matching. The algorithm emphasises more on speed and in most cases, it cannot guarantee optimal alignment of the query and base sequences. Moreover, regions with low-complexity sequences can create problems in sequence similarity searching by causing artificial hits, and are not appropriate for analysing with BLAST. Such sequences are usually determined and masked out in BLAST using DustMasker^13^ and separately analysed visually.

To overcome limitations of conventional alignment methods, a few reinforcement learning (RL) based techniques have recently been proposed in literature. Mircea et al.^14^ presented one of the first RL implementations for solving the DNA sequence alignment using Markov decision process (MDP). Jafari et al.^15^ introduced deep Q-Network (DQN) and an actor-critic algorithm in their work. The DQN model architecture was based on long short-term memory (LSTM) network which involves sequential computation to process the DNA sequence as a 1D data series. Song et al.^16^ provided a comprehensive deep RL based solution for pairwise sequence alignment, employing various pre-processing techniques such as Clustal, MUMmer, etc. to improve the alignment results, and highlighted the influence of various system parameters such as learning rate, window size, etc. on the model accuracy. Ramakrishnan et al.^17^ utilised asynchronous advantage actor critic (A3C) framework for MSA. They used a convolutional neural network (CNN) based architecture for their actor-critic model. However, this model is too complex to be applied to practical datasets with dozens of sequences and hundreds of molecules. Joeres et al.^18^ offered a comprehensive analysis of the performance of different RL algorithms for MSA, and observed that RL algorithms are typically much slower as compared to traditional solutions. However, all of these RL implementations either used very large models, which are challenging to implement on resource-constrained hardware, or or employed neural networks such as LSTMs, that are slower and currently not supported by machine learning frameworks for microcontrollers such as TensorFlow Lite^19^. In this work, we present EdgeAlign, a compact deep RL model for pairwise sequence alignment deployed on embedded edge device platforms such as the NVIDIA Jetson Nano Developer Kit and Digilent Arty A7-100T, containing Xilinx XC7A35T Artix-7 FPGA. The RL agent is modelled as a dueling DQN architecture, the number of parameters in which are optimised using a technique presented in this work for neural network model size reduction using AutoML^20^ for deep RL applications. The results are benchmarked on sequences from the publicly available Influenza sequences at the National Center for Biotechnology Information (NCBI) Virus Data Hub. EdgeAlign implementation source code is freely available on GitHub^21^.

## Methods

### Pairwise sequence alignment using deep reinforcement learning

Deep RL is a very popular topic among machine learning enthusiasts and researchers, with varied range of applications such as robotics, autonomous driving, navigation, human-like behavior in AI, system modeling, etc.^22^. Unlike supervised learning, where the feedback provided to an agent (model) is correct set of actions for performing a task (classification), RL employs an agent to learn in an interactive environment by trial and error, using feedback from its own actions and experiences in form of rewards and punishments as signals for positive and negative behaviour, respectively. RL also differs from unsupervised learning in terms of the goals of the model. The goal of an RL model is to devise a suitable action model that maximises the cumulative reward of the agent for a wide range of test cases, while the goal in unsupervised learning is to find similarities and differences (anomalies) between data points. Most conventional RL algorithms are based on a tabular approach for choosing the best actions by observing the current environment or states. However, in many practical decision-making scenarios, the states are high-dimensional and often difficult to be modelled by traditional RL algorithms. On the other hand, deep neural networks are known to be excellent function approximators^23^, and therefore more suitable for modelling complex systems.

For the pairwise sequence alignment problem addressed in this paper, we have employed a deep Q-network (DQN) agent to estimate the scoring strategy for taking appropriate action in a given state. The DQN architecture explicitly separates the representation of state values and state-dependent action advantages via two separate streams. We have also used a dueling neural network architecture which consists of two streams that represent the value and advantage functions, while sharing a common convolutional feature-learning module^24^. The two streams are combined via a special aggregating layer to produce an estimate of the state (*s*)-action (*a*) value function *Q*(*s*, *a*). The key motivation behind this architecture is that it is not necessary to know the value of each action at every step; only the most advantageous action needs to be determined. The DNA sequences to be aligned were represented as 3D images, which were processed by the network. Each type of nucleotide (A, C, G, T) was converted to a $3 \times 3$ pixel square with CMYK color representation. To separate the left, right, top, and bottom ends of the sub-sequences, $3 \times 3$ empty pixels were padded. During training, the nucleotide representation was converted into RGB format for dimensionality reduction and ease of visualisation. The agent operates upon a finite-sized window i.e. subset of the sequences to be aligned, and takes appropriate action (forward, insertion or deletion) for the first nucleotide in each sub-sequence based on the information contained in the window. Each action incurs a suitable reward or penalty, as discussed below. The window was accordingly updated and slid over the length of the entire sequence, and the process was repeated to complete the alignment task. Since the window size is fixed, the number of computations, and therefore hardware resources required, are fixed for any sub-alignment task. This is highly advantageous for larger sequences, as they can be processed using finite pool of resources.

**Figure 1:**
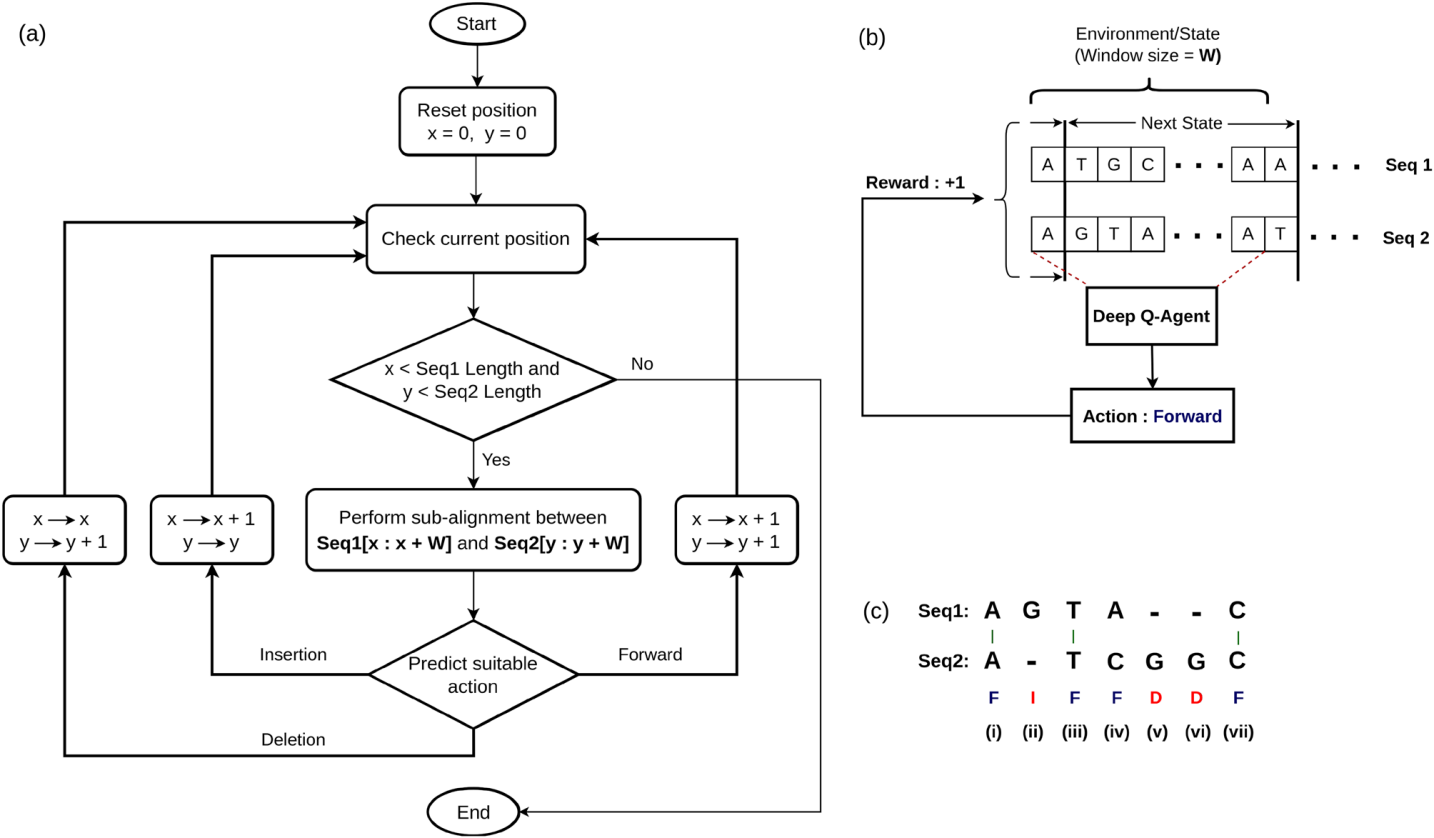
(**a**) Overview of EdgeAlign algorithm, operated on two sequences (Seq1 and Seq2). *x* and *y* denote the current nucleotide indices for the first and second sequence, respectively, and *W* denotes the size of the window which is slid over the sequences, starting from the current nucleotide indexes. The next action is predicted using a deep neural network-based RL agent. (**b**) The deep Q network RL agent operates upon sub-sequences in the current window (environment) and chooses a suitable action using the RL agent, earning a reward (or penalty) accordingly. Once the reward and action are determined, the agent moves on to the next window, which is updated based on the current action. The overall sequence alignment is performed by repeating these sub-alignments, until the agent reaches the end of either of the sequences. (**c**) The EdgeAlign RL agent can choose among the possible actions: forward (F), insertion (I), and deletion (D) to maximise the obtained rewards in an alignment task.

Figure 1a illustrates the dataflow in EdgeAlign algorithm. Analysis of data in a window (of size *W*) starts from a prescribed starting point of both sequences, and the window is slid till it reaches the end of either of the sequences. *x* and *y* represent the current nucleotide indices for the first (Seq1) and second (Seq2) sequence, respectively. Figure 1b illustrates how the RL environment/state is formed and updated based on the predicted action. Figure 1c shows the 3 possible discrete actions of the RL agent in the pairwise sequence alignment problem. In case of forward action, the agent simply shifts the window to the right by one position for both the sequences, corresponding to finding a a match at the current index. The ultimate result could be either a true nucleotide match e.g. (*i*, *iii*, *vii*) or a mis-match e.g. (*iv*), however, the agent simply decided to go forward. In case of insertion, the agent inserts a gap in the second sequence e.g. (*ii*) and shifts the window to the right by one position for the first sequence, thus corresponding to insertion of an extra nucleotide in the first sequence and a gap in the second sequence. Similarly, in case of deletion, the agent inserts a gap in the first sequence e.g. (*v*) and shifts the window to the right by one position for the second sequence. These 3 actions will correspond to 3 different Q-values at the output of the deep Q-network.

### Alignment score and reward policy

EdgeAlign aligns two given sequences in such a way that we obtain maximum number of matches between the sequences. However, alignments with too many gaps or mismatches are not desirable. A commonly used metric to characterise the quality of the alignment is the BLAST alignment score. This rule-based metric assigns a numerical score to any alignment, with a higher score indicating better alignment. This alignment score is computed by assigning a value to each aligned pair of nucleotides and then adding these values over the length of the sequence. For nucleotide alignments, the default BLAST options^25^ use a reward of $+\,1$ for each match and a penalty of $-\,3$ for each mismatch. The creation or opening of a gap in an alignment results in a negative gap-opening penalty of $-\,5$, with each extension of an existing gap incurring a lesser penalty of $-\,2$. The BLAST alignment score should also influence the reward system used for training the agent, since the alignment results from the RL tool would be evaluated using this scoring mechanism. In EdgeAlign implementation, the rewards (i.e. penalties) for mismatch $(-\,0.6)$, gap opening $(-\,1)$, and gap extension $(-\,0.4)$ were down-scaled and chosen to be in the range of $(-\,1, 1)$. The reward for a match was chosen to be $+\,1$ in order to introduce a bias towards obtaining more matches. As an example alignment shown in Fig. 1c, there are 3 nucleotide matches (*i*, *iii*, *vii*), 1 mis-match (*iv*), 2 gap openings (*ii*, *v*) and 1 gap extension (*vi*). Thus, the total alignment score is the cumulative sum of the individual rewards, which in this case comes out to be $(3\times 1) + \left( 1\times (-\,0.6)\right) + \left( 2\times (-\,1)\right) + \left( 1\times (-\,0.4)\right) = 0$.

### Preprocessing

**Figure 2:**
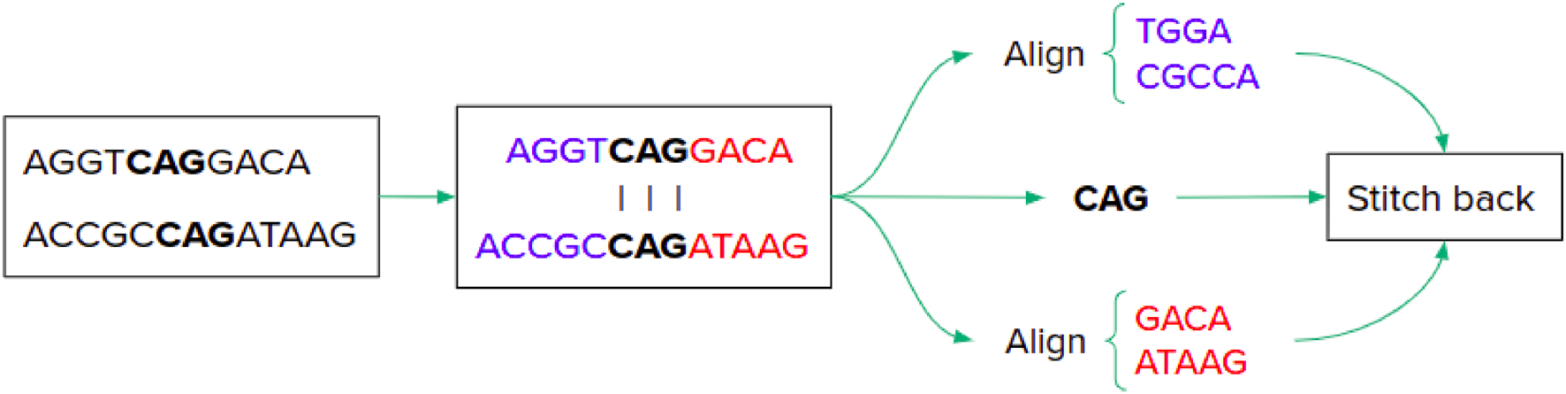
Illustration of longest common substring (LCS) preprocessing method. LCS provides the starting point for the alignment process. The forward and backward sub-alignment results are combined together to obtain the final alignment result.

EdgeAlign utilises a sliding window-based approach to employ RL for performing sequence alignment, and is therefore a sequential method wherein any decision or action once implemented for the sub-sequences in a window, cannot be modified or undone as the window slides forward. Thus, the quality of the solution (i.e. alignment score) is greatly dependent on the starting position in each sequence, because the alignment procedure is performed only in one direction. We can remove such variability by applying pre-processing techniques used in conventional alignment methods (Clustal Omega and the MUMmer), such as the longest-common substring (LCS)^16^. EdgeAlign uses this technique to find the longest substring which is common to both sequences. Using this longest-common substring (LCS) as the starting point, the alignment task is split into two sub-alignment jobs. For all sub-sequences to the left of LCS, the alignment task is performed in the backward direction. On the other hand, for all sub-sequences to the right of LCS, the alignment task is performed in the forward direction. The aligned sub-sequences from both sub-alignment jobs are stitched back to the LCS, to obtain the overall alignment solution. Figure 2 illustrates the LCS preprocessing method. The computational time complexity of the LCS algorithm for two sequences of length *m* and *n* respectively, is *O*(*mn*). Thus, it may not be feasible to use this method when working with very large sequences (e.g. human genomes). In such cases, it may be advisable to bypass this preprocessing step and only proceed with the forward alignment.

### Network architecture and training

**Figure 3:**
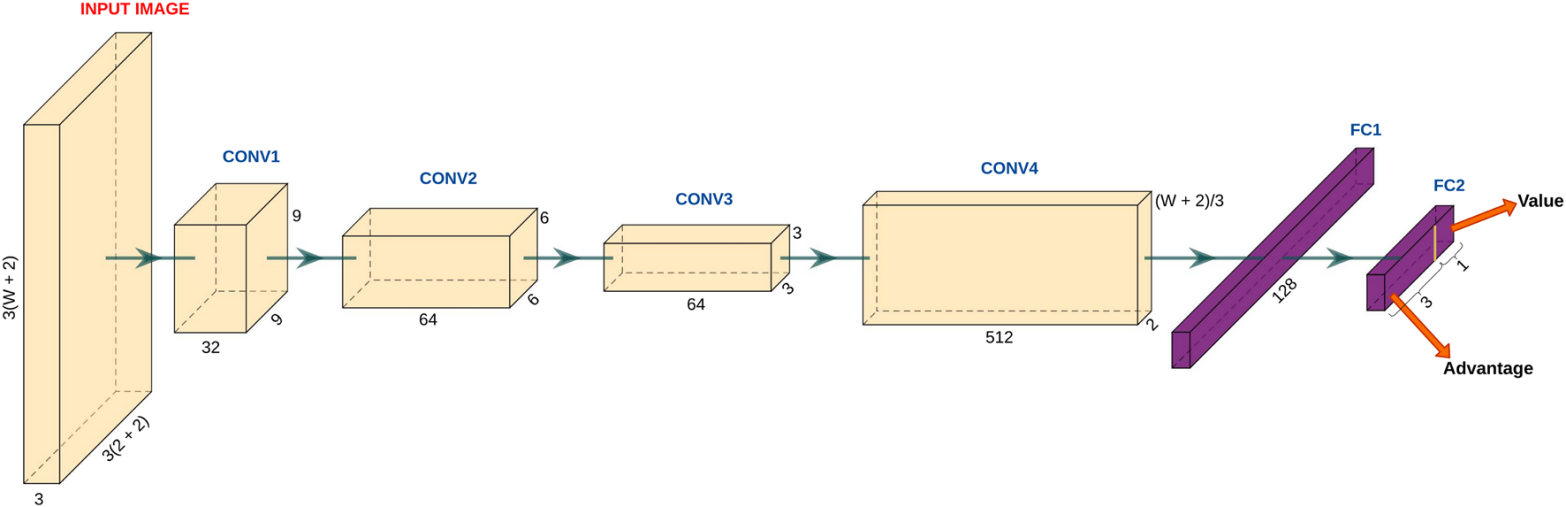
Illustration of network architecture ($W =$ window size). The filter shape at each step is represented by the dimension of the corresponding convolution block. Input to the network is an RGB image.

The DQN agent was modelled using a CNN with a dueling network architecture. By representing the sequences as images, the task of determining reward and action for sub-alignment within a window is posed as an image classification problem. CNN is the most widely used network architecture for image classification tasks due to advantages it affords in terms of dimensionality reduction, feature sharing, and pattern finding. Using the dueling method, the RL agent learns the scores of the states (value) and actions separately, thus helping improve the stability and convergence of the learning process. Figure 3 and Table 1 show the detailed network architecture and the model parameters, respectively. We used 4 convolutional block layers with ReLU output activation for identifying the features in the input image, each with an increasing number of filters and granularity. Since we require a fixed size and flattened output at the end of the CNN operations, the filter shape of the last convolutional layer depends on the window size. The last layer of the model has 4 output neurons with linear activation corresponding to the state value and advantage values for 3 different actions. The value and advantage outputs are aggregated to yield the Q-values. In the implementation presented here, a single fully-connected layer (FC2) is used for computing both the value and advantage outputs. We could also design different networks for handling the value and advantage output streams separately, or introduce more fully-connected layers for computing the output of each stream in a branched manner. However, this would result in an increase in the number of parameters in the model, and therefore greater resource consumption and inference time. For demonstrating the feasibility of our method, we have limited our study to a single fully-connected layer at the output.

**Table 1:** Network architecture ($W =$ window size). ${\lceil {*}\rceil }{~}$ denotes the ceiling function.

Layer	Input size	Filter shape	Stride	Padding
Conv1	$3(W+2) \times 12 \times 3$	$9 \times 9 \times 32$	$3 \times 3$	Same
Conv2	$(W+2) \times 4 \times 32$	$6 \times 6 \times 64$	$3 \times 3$	Same
Conv3	${{\lceil {(W+2)/3}\rceil }} \times 2 \times 64$	$3 \times 3 \times 64$	$1 \times 1$	Same
Conv4	${{\lceil {(W+2)/3}\rceil }} \times 2 \times 64$	${{\lceil {(W+2)/3}\rceil }} \times 2 \times 512$	$1 \times 1$	Valid
FC1	512	128	–	–
FC2	128	4	–	–

**Figure 4:**
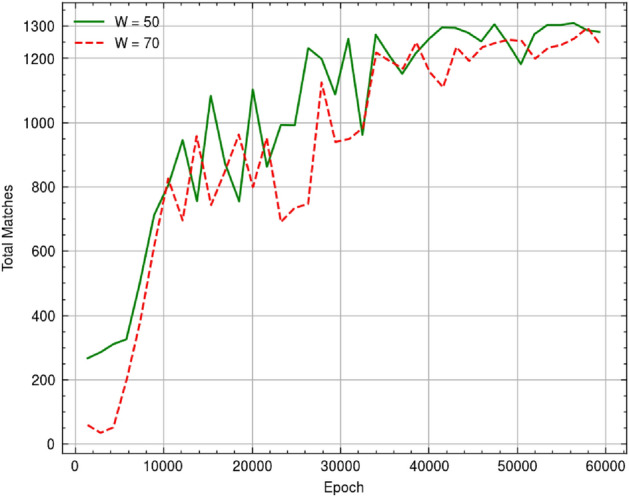
Increasing trend observed for the number of matches obtained by the EdgeAlign RL agent during training for window sizes of 50 and 70.

The nature of the pairwise sequence alignment problem makes it easy to generate artificial DNA sequence data with controllably introduced mutations for training the RL agent. These sequences must be generated using a definite rule and should be able to mimic various mutations such as SNP (single nucleotide polymorphism), insertions and deletions (InDels). For this purpose, we generated various random sequences, and their corresponding paired sequence through introducing mutations using JC69 model to create SNP mutations^26^, and Zipfian distribution-based InDel length model for generating InDels^27^. This functionality was implemented using Python as this allowed us to easily integrate the sequence generator with our RL environment. We used Keras-RL^28^ as the RL framework for training the dueling DQN agent, implemented using *Dueling DQNAgent* from the Keras-RL framework with a *Greedy* policy for determining suitable actions. Figure 4 shows increasing trend in number of matches with increasing epochs, obtained by the RL agent for 2 different window sizes (50 and 70) during a training session for sequences of length 1500.

### Model size reduction using AutoML

It is important that the model size for pairwise sequence alignment using EdgeAlign be minimal, so that the trained model could be implemented on an edge device. For a window size of 50, the implementation described in the previous section (Table 1) comprises of approximately 1.36 M parameters, and is therefore not suitable for edge implementation. Pruning and quantisation are commonly employed methods for performing model compression^29,30^. However, it is often cumbersome to evaluate the importance of each neuron in the network for pruning, and usually requires manual fine-tuning. Another method for model compression is knowledge distillation^31^, wherein a pre-defined smaller network is trained using a fully-trained larger network as ground truth. This approach is similar to transfer learning, and requires setting up a fully-trained network. Quantisation is a well-known approach for compressing a model, by representing the model parameters using 8-bit integers instead of floating point numbers. This provides large savings in memory footprint for storing the model parameters, albeit with deteriorated model performance due to reduction of numeric precision and resolution. In pairwise sequence alignment, this is undesirable, because the alignment is performed in a sequential and unidirectional manner, and therefore errors in each alignment task for sub-sequences accumulate and degrade the overall model performance.

For a given labelled dataset, neural architecture search (NAS)^32^, is a suitable technique for automating the design of neural network architectures within a desired set of constraints e.g. maximum allowed model size, number of operations, latency etc. NAS only requires the search-space constraints to be specified, and a labelled dataset to be provided to the NAS tool for generating an optimal model that provides best performance under the user-specified constraints on that dataset. In this paper, we have utilised the power of NAS using AutoKeras, an open-source tool for performing the model size reduction. AutoKeras is an open-source AutoML system based on the Keras framework^33^. It provides a simple and effective approach for automatically finding top-performing models for a wide range of predictive modelling tasks, including tabular or structured classification and regression datasets.

The technique of AutoML is best suited for supervised learning applications with comprehensive, labelled datasets. On the other hand, RL frameworks rely on learning underlying rules in a training environment and do not require a labelled dataset. Moreover in the sequence alignment problem, the next pair of sub-sequences to be analysed (once the window is slid) will be decided by the current action predicted by the partially-trained model. Hence, the data is also influenced by the model, and it is therefore difficult to incorporate conventional NAS techniques for model size reduction in EdgeAlign. However, using the conventional deep-Q network presented in the previous section (e.g. model with window size of 50 and approximately 1.36 M parameters), it is possible to replicate the performance of this RL agent with a smaller model generated with AutoKeras. This is achieved by running the sequence alignment model on any given pair of input sequences and saving the intermediate states (window of sub-sequences) separately. Further, the pre-trained network is used to generate a labelled dataset, which consists of predicted actions corresponding to each of these input states. Next, we can feed this generated dataset to the AutoKeras framework for finding an optimal (i.e. reduced size) model architecture with far lesser number of parameters. In theory, this optimal model should be able to replicate the performance of the pre-trained network. This process can be summarised using the following equations:

1 $$f(s,\theta ) \longleftrightarrow \text {AutoKeras}\longleftrightarrow f^{\prime}(s,\theta ^{\prime})$$

2 $$f(s,\theta ) \approx f^{\prime}(s,\theta ^{\prime}), |\theta ^{\prime}|\ll |\theta |$$

where $\theta$ and $\theta ^{\prime}$ are the model parameters of the pre-trained and the reduced size model, respectively, *s* represents the input states, and *f* and $f^{\prime}$ represent the neural network functions for the pre-trained and the reduced size model, respectively. The actions predicted by the pre-trained network are used to assign labels, and used for training the network generated by AutoKeras. For reducing the input dimension, we also represented each individual nucleotide with a $2\times 2$ pixel square for the reduced size model. Using this technique, the pre-trained network for window size of 50 with approximately 1.36 M parameters was reduced to a network with approximately 98 k parameters using AutoKeras. Our proposed technique is fully automated and does not require any manual fine-tuning or prior information regarding the reduced size model architecture, unlike quantisation or pruning. The detailed network architecture of the reduced size model, benchmarking results and inference timing as compared to the pre-trained network are discussed in the next section.

## Results and discussion

**Figure 5:**
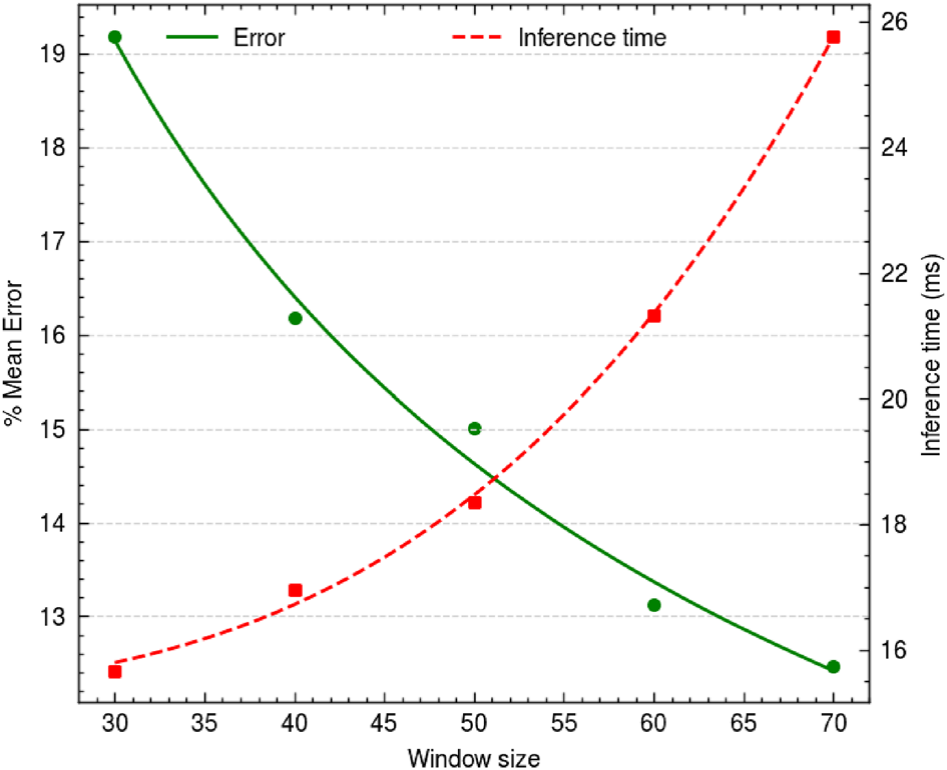
Benchmarking results on the NCBI Virus Data Hub influenza dataset.

We evaluated the performance of EdgeAlign on publicly available Influenza sequences at the National Center for Biotechnology Information (NCBI) Virus Data Hub^34^. The NCBI online tool also provides the BLAST alignment result for a chosen pair of sequences. The objective is to compare the alignment score obtained by the NCBI online tool and EdgeAlign. For fair benchmarking, we analysed the following error metric (*E*) for a given pair of input sequences:

3 $$E = \left\{ {\begin{array}{*{20}l} {0,} & {{\text{R}} \ge {\text{G}}} \\ {\frac{{G - R}}{G}} & {{\text{R}} < {\text{G}}} \\ \end{array} } \right.$$

where *R* denotes the alignment score obtained using EdgeAlign, and *G* denotes the alignment score obtained using the NCBI online tool (ground truth). We evaluate the mean error for the target dataset (*N* pair of sequences): $\frac{1}{N}\sum _i E_i$. This evaluation was performed on a dataset comprising of 40 randomly chosen sequences, with 5 different window sizes. Further, we deployed the DQN network on the NVIDIA Jetson Nano Developer Kit and obtained the inference time required for predicting a single action. The NVIDIA Jetson Nano Developer Kit is a small (69$\times$45 mm), powerful computer with Jetson Nano 2 GB module (128 core NVIDIA Maxwell GPU, Quad-core Arm Cortex-A57 MPCore processor, 2 GB LPDDR4 Memory) that consumes less than 5 W power and is suitable for edge-computing applications. Table 2 and Fig. 5 summarise the benchmarking results on the NCBI Virus Data Hub dataset. As evident from the results, the mean error decreases with an increase in the window size. With a larger window, the agent can observe more sequences and take a more informed action. This increased awareness leads to better results as the chosen actions are more reliable for securing higher rewards over the length of the sequence. However, it also increases resource consumption and consequentially, the inference time. These trade-offs must be accounted for while choosing the window size and device for edge implementation. For this study, we performed model size reduction using AutoKeras for 2 different window sizes: 50 and 70. Table 3 shows the resulting network architecture and the model parameters obtained for a window size of 50. The benchmarking results on the NCBI Virus Data Hub influenza dataset and the inference time and mean error obtained from the optimised models are shown in Table 4.

**Table 2:** Benchmarking results of DQN networks on the NCBI Virus Data Hub influenza dataset.

Window size	Number of parameters	Inference time (ms)	Mean error (%)
30	906,116	15.67	19.19
40	1,102,724	16.96	16.18
50	1,364,868	18.36	15.01
60	1,561,476	21.33	13.12
70	1,758,084	25.77	12.47

**Table 3:** Network architecture of the optimal AutoKeras model (window size: 50).

Layer	Input size	Filter shape	Stride	Padding
Conv1	$100 \times 4 \times 3$	$3 \times 3 \times 32$	$1 \times 1$	Same
Conv2	$100 \times 4 \times 32$	$3 \times 3 \times 32$	$1 \times 1$	Same
Max pooling	$100 \times 4 \times 32$	$2 \times 2$	$2\times 2$	–
Conv3	$50 \times 2 \times 32$	$3 \times 3 \times 64$	$1 \times 1$	Same
Conv4	$50 \times 2 \times 64$	$3 \times 3 \times 32$	$1 \times 1$	Same
Max pooling	$50 \times 2\times 32$	$2 \times 2$	$2 \times 2$	–
Flatten	$25 \times 1 \times 32$	–	–	–
FC1	800	64	–	–
FC2	64	4	–	–

**Table 4:** Benchmarking results of pre-trained DQN and optimised model generated using AutoKeras on the NCBI Virus Data Hub influenza dataset. The computations were performed on the NVIDIA Jetson Nano Developer Kit with floating point precision.

Window size	50		70	
Model	Pre-trained DQN	AutoKeras	Pre-trained DQN	AutoKeras
Number of parameters	1,364,739	98,628	1,758,084	119,108
Inference time (ms)	18.36	2.32	25.77	9.82
Mean error (%)	15.01	17.98	12.47	11.79

The reduced size model generated with AutoKeras is suitable for edge deployment. We deployed and evaluated performance of the model with window size of 50 on some representative edge platforms. To evaluate performance on a low-cost embedded platform with very limited resources, we chose the 32F746GDISCOVERY Discovery kit containing the STMicroelectronics Arm Cortex-M7-core-based STM32F746NG microcontroller with 1 MB of Flash memory and 340 kB of RAM. The AutoKeras generated model was exported as a TensorFlow Lite model (.tflite), and converted to a header file (.h) that can be processed by TensorFlow Lite for Microcontrollers framework^19^. During run-time, we first read both sequences from a host computer using an UART port. Next, we invoke the TesorFlow Lite model and perform sequence alignment on the edge device. Finally, we send the list of actions computed by the model back to the host computer via the same UART port. A pySerial (Python serial port extension) link was setup on the host computer interface to navigate this communication. Presently, TensorFlow Lite for Microcontrollers does not support fixed-point precision. Using 8-bit integer representation resulted in a model of size approximately 100 kB for window size 50. However, as expected the reduction of numeric precision resulted in large errors, with approximately 70% reduction in number of matches found as compared to the pre-trained DQN network. Therefore, it is necessary to use floating-point precision for representing the model, and hence a microcontroller device cannot be used as the edge platform.

We then evaluated the model performance on a more capable embedded system, namely a field programmable gate array (FPGA) device. For this purpose, we chose the Digilent Arty A7-100T, containing Xilinx XC7A35T Artix-7 FPGA. The program instructions and model data are stored in the onboard DDR3 memory, and the block RAM (BRAM) is reserved for implementing custom hardware accelerator IPs. We observed the inference time for predicting a single action with the AutoKeras model of window size 50 to be 2.05 s for 100 MHz clock. The mean error on the FPGA remains same as before since all the computations were performed in floating-precision. To improve the performance, we implemented a CNN-layer accelerator to perform the convolution operation based on the row-stationary approach, inspired by the Eyeriss architecture^35^, which is an energy-efficient reconfigurable accelerator for deep convolutional neural networks. Such a design allows us to maximise parallelism and data reuse in the convolution operation. This IP was designed and implemented using the Xilinx Vivado HLS tool (version 2021.2), and was integrated with the on-chip MicroBlaze (32-bit RISC soft processor) core using the Xilinx Vivado tool. The reference code and detailed documentation for porting TensorFlow Lite model to MicroBlaze core is available on GitHub^36^. The corresponding TFLite kernels were also modified to invoke the custom IPs. This resulted in a speed-up factor of 2 X, and inference time of 1 s for 100 MHz clock. While this is considerably slower than the low-cost GPU (NVIDIA Jetson Nano Developer Kit), the ability to deploy sequence alignment algorithms on embedded systems such as microcontrollers and FPGAs presented in this work enables the use of such platforms for low-cost bioinformatics applications. Table 5 shows the resource utilisation for the FPGA implementation, with and without the usage of custom accelerator IPs. For interested readers, a more detailed report about the hardware implementation and design of custom accelerators used in this work is provided on GitHub^37^.

A common feature of deep reinforcement learning-based implementations for sequence alignment reported in literature^15,17,18^, is that none of them are deployed on edge devices, and therefore relevant performance metrics such as inference time (per action) are not reported. The implementation reported by Jafari et al. uses a model with > 33 K parameters^15^, and Ramchalam et al. reported a model with > 0.19 M parameters^17^. Model size was not reported for the implementation shown by Joeres et al.^18^. All these implementations were based on multiple sequence alignment and used SP score (sum-of-pairs) as the alignment scoring metric. The pairwise sequence alignment implementation by Song et al.^16^ used a model with > 1.38 M parameters, with a simulation based inference timing of 14 ms. The alignment score was evaluated using Exact Matches metric. The EdgeAlign model uses BLAST score as the alignment score, and the model size of approximately 98 k parameters and inference time of 2.32 ms per action (AutoKeras with window size of 50, implemented on NVIDIA Jetson Nano Developer Kit) are better than the implementation reported by Song et al.^16^.

**Table 5:** Resource utilisation for the Arty A7-100T FPGA implementation.

Resources in Arty A7-100T FPGA	36kbit BRAM	DSP slices	Flip-flop	LUT
Maximum available	135	240	126,800	63,400
Without hardware acceleration	13	5	12,922	11,363
With hardware acceleration	51	89	29,012	25,513

## Conclusion

In summary, this work presents a method to develop compact deep RL models using AutoML for performing pairwise DNA sequence alignment on embedded edge devices. We has demonstrated a novel method based on AutoML for reducing model size for deep reinforcement learning, while preserving floating-point precision for greater accuracy. The models thus produced have an order of magnitude lesser number of trainable parameters compared to deep RL models designed using conventional DQN architecture, and the use of Keras-RL library further streamlines the design and training process and enables easy debugging of the code. EdgeAlign, the RL-based sequence alignment tool presented in this work, is capable of processing sequences of arbitrary lengths with fixed resource utilisation, albeit with a trade-off in inference time for longer sequences. The performance results for EdgeAlign are benchmarked using the publicly available NCBI Virus Data Hub dataset to evaluate the trade-off between accuracy and throughput. Additional improvements may focus on further optimisation of the reward system and model architecture, to improve inference time and reduce resource utilisation. The window size could also be made adaptable to account for diversity in the input DNA sequences i.e., if the input sequences are less diverse, we could use a shorter window size. This parameter can be adjusted during run-time, based on live performance of the model. The AutoML based model size reduction technique can also be used in various other applications where inference time is a critical specification, such as visual applications including drone navigation, simultaneous localisation and mapping (SLAM), beamforming, etc.

## Data Availability

The sequences used for benchmarking EdgeAlign performance were obtained from the publicly available Influenza sequences at the National Center for Biotechnology Information (NCBI) Virus Data Hub^34^. FASTA format files of the sequences analysed are available on GitHub^21^: https://github.com/aryanlall11/EdgeAlign/tree/master/Influenza.

## References

[1] Mattick JS (2014). The impact of genomics on the future of medicine and health. Med. J. Aust..

[2] Jain M, Olsen HE, Paten B, Akeson M (2016). The Oxford Nanopore MinION: Delivery of nanopore sequencing to the genomics community. Genome Biol..

[3] Needleman SB, Wunsch CD (1970). A general method applicable to the search for similarities in the amino acid sequence of two proteins. J. Mol. Biol..

[4] Larkin MA (2007). Clustal W and Clustal X version 2.0. Bioinformatics.

[5] Edgar RC (2004). MUSCLE: Multiple sequence alignment with high accuracy and high throughput. Nucleic Acids Res..

[6] Katoh K, Standley DM (2013). MAFFT multiple sequence alignment software version 7: Improvements in performance and usability. Mol. Biol. Evol..

[7] Sievers F (2011). Fast, scalable generation of high-quality protein multiple sequence alignments using Clustal Omega. Mol. Syst. Biol..

[8] Chao K-M, Pearson WR, Miller W (1992). Aligning two sequences within a specified diagonal band. Bioinformatics.

[9] Camacho C (2009). BLAST+: Architecture and applications. BMC Bioinform..

[10] Marçais G (2018). MUMmer4: A fast and versatile genome alignment system. PLoS Comput. Biol..

[11] NCBI, B. L. A. S. T. Basic local alignment search tool—NCBI. https://blast.ncbi.nlm.nih.gov/Blast.cgi.

[12] McGinnis S, Madden TL (2004). BLAST: At the core of a powerful and diverse set of sequence analysis tools. Nucleic Acids Res..

[13] Morgulis A, Gertz EM, Schäffer AA, Agarwala R (2006). A fast and symmetric DUST implementation to mask low-complexity DNA sequences. J. Comput. Biol..

[14] Mircea, I.-G., Bocicor, I. & Czibula, G. A reinforcement learning based approach to multiple sequence alignment. In *International Workshop Soft Computing Applications* 54–70 (Springer, 2016).

[15] Jafari R, Javidi M M, Kuchaki Rafsanjani M (2019). Using deep reinforcement learning approach for solving the multiple sequence alignment problem. SN Appl. Sci..

[16] Song Y-J, Ji DJ, Seo H, Han GB, Cho D-H (2021). Pairwise heuristic sequence alignment algorithm based on deep reinforcement learning. IEEE Open J. Eng. Med. Biol..

[17] Ramakrishnan, R. K., Singh, J. & Blanchette, M. RLALIGN: A reinforcement learning approach for multiple sequence alignment. In *2018 IEEE 18th International Conference on Bioinformatics and Bioengineering (BIBE)* 61–66 (IEEE, 2018).

[18] Joeres, R. Multiple sequence alignment using deep reinforcement learning. SKILL 2021 (2021).

[19] David R (2021). TensorFlow Lite Micro: Embedded machine learning for TinyML systems. Proc. Mach. Learn. Syst..

[20] He X, Zhao K, Chu X (2021). AutoML: A survey of the state-of-the-art. Knowl. Based Syst..

[21] Lall, A. EdgeAlign. https://github.com/aryanlall11/EdgeAlign (2022).

[22] Li, Y. Deep reinforcement learning: An overview. arXiv preprint arXiv:1701.07274 (2017).

[23] Hornik K (1991). Approximation capabilities of multilayer feedforward networks. Neural Netw..

[24] Wang, Z. *et al.* Dueling network architectures for deep reinforcement learning. In *International Conference on Machine Learning* 1995–2003 (PMLR, 2016).

[25] Manual, A. N. B. C. L. A. U. Appendices—ncbi blast command line applications user manual. https://www.ncbi.nlm.nih.gov/books/NBK279684/.

[26] Jukes TH, Cantor CR (1969). Evolution of protein molecules. Mamm. Protein Metab..

[27] Qian B, Goldstein R A (2001). Distribution of indel lengths. Proteins Struct. Funct. Bioinform..

[28] Plappert, M. keras-rl. https://github.com/keras-rl/keras-rl (2016).

[29] Liang T, Glossner J, Wang L, Shi S, Zhang X (2021). Pruning and quantization for deep neural network acceleration: A survey. Neurocomputing.

[30] Deng L, Li G, Han S, Shi L, Xie Y (2020). Model compression and hardware acceleration for neural networks: A comprehensive survey. Proc. IEEE.

[31] Gou J, Yu B, Maybank SJ, Tao D (2021). Knowledge distillation: A survey. Int. J. Comput. Vis..

[32] Ren P (2021). A comprehensive survey of neural architecture search: Challenges and solutions. ACM Comput. Surv. (CSUR).

[33] Jin, H., Song, Q. & Hu, X. Auto-Keras: An efficient neural architecture search system. In *Proceedings of the 25th ACM SIGKDD International Conference on Knowledge Discovery and Data Mining* 1946–1956 (ACM, 2019).

[34] Hatcher EL (2017). Virus variation resource—Improved response to emergent viral outbreaks. Nucleic Acids Res..

[35] Chen Y-H, Krishna T, Emer JS, Sze V (2016). Eyeriss: An energy-efficient reconfigurable accelerator for deep convolutional neural networks. IEEE J. Solid-State Circuits.

[36] Lall, A. TFLite-Micro-Accelerator. https://github.com/aryanlall11/TFLite-Micro-Accelerator (2022).

[37] Lall, A. EdgeAlign Hardware Implementation. https://github.com/aryanlall11/EdgeAlign/blob/master/DDP_Report_17D070053.pdf (2022).

